# Interpreting the drop: concordance and disparity between a new point-of-care assay and laboratory intraoperative parathyroid hormone testing in parathyroid surgery

**DOI:** 10.1186/s12902-025-02112-x

**Published:** 2026-01-13

**Authors:** Devanshu Kwatra, William Wakeford, Munira Ally, Julia Dowsett, Anant Patel, Ahmad Moinie, George Mochloulis, Panagiotis A. Dimitriadis

**Affiliations:** 1 Department of ENT, Lister Hospital, Stevenage, UK; 2 Department of Clinical, Pharmaceutical and Biological Sciences, University of Hertfordshire, College Lane Campus, Hatfield, Hertfordshire, AL10 9AB UK

**Keywords:** Parathyroid hormone (PTH), Intraoperative PTH (IOPTH), Parathyroidectomy, Point-of-care (POC), NBCL CONNECT

## Abstract

**Background:**

To evaluate the concordance of the NBCL CONNECT Point-of-Care (POC) Intraoperative Parathyroid Hormone (IOPTH) assay with a Central Laboratory standard, with a focus on percentage drops and the usefulness of POC testing in parathyroid surgeries.

**Methodology:**

In this prospective single-centre study (Nov 2023-May 2025), blood samples were drawn from patients undergoing parathyroidectomy for simultaneous analysis on the NBCL CONNECT POC assay and the central laboratory platform. The timing of the blood sample would typically be at induction of anaesthesia, 5 min and 10 min post-excision. Statistical analyses were performed using Python with the SciPy and Matplotlib libraries. Wilcoxon signed-rank test was used to determine the presence of a systematic difference between the two assays. Bland-Altman analysis was performed to calculate the mean difference and the 95% limits of agreement. Pearson correlation coefficient was used to evaluate the linear relationship between the two methods.

**Results:**

Fifty-seven consecutive patients were identified and included in this study. No significant difference was found at baseline (*p* = 0.943) or 5 min (*p* = 0.098). However, a statistically significant difference emerged at 10 min (*p* = 0.003). Bland-Altman analysis revealed wide limits of agreement at all time points and correlation between the methods was poor at the 10-minute mark (*r* = 0.11). The mean percentage PTH drop reported by NBCL CONNECT was significantly greater than the laboratory values at both 5 min (58.6% vs. 49.0%, *p* = 0.001) and 10 min (51.2% vs. 36.2%, *p* = 0.009).

**Conclusion:**

The NBCL CONNECT provides a reliable baseline PTH measurement but consistently reports a larger percentage drop post-excision. Surgeons using this assay must be aware of its specific characteristics and the potential for both false-positive and false-negative results to ensure optimal surgical outcomes.

**Clinical trial number:**

Not applicable.

## Background

Parathyroidectomy is the definitive treatment for Primary Hyperparathyroidism (PHPT), offering a cure in approximately 95% of patients [1]. With single adenomas accounting for up to 85% of cases, the surgical approach has evolved from the traditional bilateral neck exploration (BNE) to a more focused, minimally invasive parathyroidectomy (MIP), which is now the recommended approach for localised disease [2, 3].

The development of intraoperative parathyroid hormone (IOPTH) monitoring has been described as a Copernican revolution in parathyroid surgery by Gioviale et al. (2016) [1]. The principle relies on the short 2–4 min half-life of intact PTH; a significant drop in its level after gland excision provides immediate biochemical confirmation that the source of PTH hypersecretion has been removed. For the surgeon, this transforms the procedure by providing real-time data to confirm surgical success and avoid unnecessary further dissection in cases of single gland disease [1]. The most widely adopted protocol to measure this drop is the Miami criterion, which defines surgical success as a >50% drop from the baseline/ highest pre-excision PTH level, 10 min after gland excision [4].

Historically, IOPTH testing required sending blood samples to a laboratory, a process fraught with logistical challenges, such as transport delays and variable turnaround times, which could significantly prolong the duration of surgery. The advent of point-of-care (POC) testing, or point-of-surgery testing, has tried to resolve these issues. Modern POC systems utilise portable analysers that employ techniques like immunochemiluminescence to deliver results within minutes, in the operating theatre itself [1]. Advanced systems, such as the NBCL CONNECT assay evaluated in this study, further streamline the process by using a single-step immunoassay that can be performed on whole blood, eliminating the need for a centrifuge or specialised technicians [2].

The fundamental difference between NBCL point-of-care PTH testing and the Roche Cobas e-601 (Elecsys) system, which we use, lies in the sample used and signal generation, which dictates the speed of testing. The NBCL device uses whole blood, eliminating the time-consuming centrifugation step required by the Cobas system, which needs processed serum or plasma. Mechanistically, both use a sandwich immunoassay with magnetic particles, but the final detection step differs; the NBCL test uses a chemical trigger solution to produce a chemiluminescent signal, whereas the Roche Elecsys system uses an electrical voltage to initiate an electrochemiluminescent reaction. These distinctions allow the NBCL analyser to provide rapid results in minutes at the patient’s side, while the Cobas system involves a more complex, lab-based workflow with a longer turnaround time.

Despite these advancements, the routine use of IOPTH as an aid in parathyroid surgeries has been debated. The UK’s National Institute for Health and Care Excellence (NICE) cited cost-effectiveness concerns in its 2019 guidance, a stance that has been directly challenged by studies demonstrating the potential cost savings of routine IOPTH use [5, 6]. More recent decision model analyses have also suggested that IOPTH use is indeed cost-effective, by reducing surgical failures and reoperation rates [7].

The aim of this study was to determine the clinical and statistical agreement between the POC assay (NBCL) and the hospital laboratory testing (gold standard) across key intraoperative time points.

Ethical approval was not required for this study as it was conducted as part of a service evaluation project which was recommended by the Trust to validate the new POC testing locally, against the gold standard test.

## Methodology

### Patient selection and pre-operative workup

All 57 patients underwent a standardised pre-operative workup, which routinely included two imaging modalities: a neck ultrasound and a Technetium-99 Sestamibi SPECT-CT scan. A few patients also underwent a Choline PET scan, if the initial two scans did not show any positive findings. The cases included in the study comprised a mix of targeted parathyroid adenoma excision and four-gland exploration depending on the scan results. All cases were discussed in a local parathyroid multidisciplinary team (MDT) meeting, where a consensus was reached before patients were offered surgery.

### Study design

This was a prospective, single-centre study conducted at Lister Hospital, Stevenage, UK. All patients undergoing parathyroidectomy for PHPT between November 2023 and May 2025 were included and were operated on by three Head and Neck Consultants in the Department.

### IOPTH protocol and assays

During surgery, peripheral venous blood samples were drawn at pre-incision, 5 min, and 10 min post-excision. For some of our patients, samples were also drawn at 15 and 20 min intervals if the first post-excision sample showed a less than 50 per cent drop, but the excised gland looked convincing of being an adenoma. After the sample was analysed in the operating theatre using the NBCL CONNECT assay, the same blood bottle was sent to the hospital laboratory (Roche Cobas platform). The processing time for the NBCL Connect assay to give results was 5 min after loading the cartridge in the machine. The results from the same sample but analysed in the central laboratory were received via email after the surgery from the POC testing coordinator. The blood results from the NBCL assay were used to guide the surgical decision making and optimise operating theatre efficiency and our institutional protocol for intraoperative PTH monitoring was followed. This protocol stipulated that subsequent blood draws could be omitted if the 5-minute post-excision sample demonstrated a PTH reduction of greater than 50% from the pre-incision baseline. It should be noted that this institutional guideline represents a modification of the strict Miami criterion, which defines surgical success as a reduction of greater than 50% in PTH levels from the highest pre-excision value, measured 10 min after the removal of a suspected hyperfunctioning parathyroid gland. We instead used a greater than 50% reduction at any point up to 20 min post excision as a positive result. During the surgical procedure, in addition to IOPTH monitoring, Near Infrared Autofluorescence (NIRAF) imaging using the Fluobeam^®^ system was routinely utilised as an adjunct to help identify parathyroid tissue.

### Objectives

The primary objective was to correlate the absolute PTH values and compare the percentage of PTH drops. The secondary objective was the characterisation of discordant results that included:

- **False positive**: A drop of > 50% PTH seen, but the patient was not cured (defined by persistent hypercalcaemia on the 4-6-week follow-up visit or a need for reoperation).
- **False negative**: Miami criteria not met, but the patient was biochemically cured (Normal Calcium and PTH at 4–6 weeks post-op).

### Statistical analysis

Statistical analyses were performed using Python with the SciPy and Matplotlib libraries.

Shapiro-Wilk test was used to check if the differences between the paired values followed a normal distribution. As the differences did not follow a normal distribution, the non-parametric Wilcoxon signed-rank test was used to determine the presence of a systematic difference between the two assays.

To provide an assessment of the clinical agreement, Bland-Altman analysis was performed to calculate the mean difference (bias) and the 95% limits of agreement. The strength and direction of the linear relationship between the two methods were evaluated using the Pearson correlation coefficient (r).

Box plots were used to compare the distribution of values at each time point and scatter plots to illustrate the degree of concordance between the assays.

A p-value of less than 0.05 was considered statistically significant for all tests.

## Results

### Patient cohort and primary comparison

A total of 57 paired PTH measurements were available for analysis at the pre-incision and 5-minute time points. This number decreased to 42 pairs at 10 min and 8 pairs at 15 min (Table 1). Blood samples were taken at 5-minute intervals post excision till a > 50% drop in the PTH level was seen (maximum till 20 min post-excision interval).

**Table 1:** PTH values (pmol/L) at different time intervals

	Pre-incision PTH (NBCL)	5 min PTH (NBCL)	10 min PTH (NBCL)	15 min PTH (NBCL)	20 min PTH (NBCL)	Pre-incision PTH (Lab)	5 min PTH (Lab)	10 min PTH (Lab)	15 min PTH (Lab)	20 min PTH (Lab)
**1**	16.3	8.63	7.64			16.54	8.75	7.71		
2	12	4.66				13.81	4.72			
3	44.2	4.77				53.62	5.45			
4	18.5	10.6	5.31			18.85	13.14	5.99		
5	15.3	3.41				24.69	4.2			
6	7.8	10.2	3.46			11.25	14.93	4.37		
7	11.4	5.34				13.44	4.84			
8	18.9	3.92				15.53	2.28			
9	23.8	11.4	13.3	8.65		21.52	8.03	4.52		
10	9.16	4.68	4.14			8.32	1.45	1.34		
11	16.3	8.61	10.7	13	9.77	12.52	8.66	7.65	7.09	6.52
12	14.1	18.2	17.7			11.27	14.11	12.42		
13	20.2	15.2	8.72			17.43	7.64	6.61		
14	12.3	12	1.36			16.38	16.33	18.55		
15	17.2	3.61	4.8			15.8	2.06	1.65		
16	13	5.54	12	4.43		13.54	4.43	2.42	2.02	
17	32.5	10.4				10.26	2.02			
18	49.91	4.21	6.19			36	8.01	6.37		
19	9.54	6.86	5.46			7.62	4.08	2.75		
20	8.5	3.7	2.22			9.87	2.87	2.45		
21	13.9	8.65	8.66	7.86		8.34	1.63	1.44	1.33	
22	13.2	32.1	11.1	6.66		13.3	35.13	12.65	7.1	
23	10.6	7.33	19.5	3.39		9.03	6.5	2.89	2.16	
24	6.81	5.45				9.25	5.48			
25	24.2	29.5	6.29			20.9	23.72	5.06		
26	13	13.1	10.5			12.49	9.79	8.63		
27	22.8	11.6	11.2			14.81	6.01	5.43		
28	22.2	8.32				17.6	6.35			
29	22.9	9.05	8.01			19.5	4.68	3.24		
30	24.9	23.1	19			7.79	1.91	3.34		
31	7.83	1.51				8.47	2.19			
32	9.42	3.23	2.09			10.9	3.98	2.73		
33	19.7	7.44	6.72			16.3	1.8	1.14		
34	18	12.5	7.59			14.27	8.22	6.42		
35	9.42	3.23	2.09			10.9	3.98	2.73		
36	34.2	41.7	37.3			15.29	7.19	2.14		
37	8.63	2.81	0.72			11.13	3.78	1.96		
38	11.1	0.63	3.35			18.91	5.43	3.62		
39	16.7	3.19				20.8	5.87			
40	57.4	20	9.78			50.45	18.41	6.89		
41	13.5	4.75				21.18	9.3			
42	12.3	1.59				15.36	3.32			
43	43.7	4.5	5.6			40.7	4.94	4.9		
44	69.5	25	20.3			77.1	17.42	12.32		
45	8.82	5.11	3.17			9.41	2.92	1.86		
46	8.88	5.62	3.14			10.21	5.47	2.24		
47	10.9	5.3	4			14.64	5.68	4.38		
48	6.28	6.61	6.39	4.38		10.84	8.03	8.31	4.43	
49	11	7.74	7.52	5.48		8.43	3.68	2.21	1.2	
50	17.3	8.38	5.56			22.37	11.06	8.31		
51	12	5.54	3.96			15.11	7.38	5.48		
52	6.22	2.32				7.77	2.65			
53	12.3	3.9				12.28	3.79			
54	11	6.65	4.92			10.5	6.93	4.42		
55	5.45	9.49	7.21	3.28	1.68	8.64	8.35	8.88	1.88	1.45
56	6.37	0.88				5.87	1.1			
57	7.01	8.84	1.67			10.25	10.48	1.73		

The results, summarised in Table 2, show no statistically significant systematic difference between the NBCL and Lab values at the pre-incision (*p* = 0.943) and 5-minute (*p* = 0.098) time intervals. However, a statistically significant difference emerged at 10 min (*p* = 0.003) and 15 min (*p* = 0.039), with the NBCL assay demonstrating a positive bias.

**Table 2:** Comparison of PTH measurements using Wilcoxon Signed-Rank test

Point of testing	Number of paired samples	Wilcoxon test *p*-value	Statistically significant difference (*P* < 0.05)
Pre-incision	57	0.943	No
5 min	57	0.098	No
10 min	42	0.003	Yes
15 min	8	0.039	Yes

The Pearson correlation coefficient indicated a strong, significant linear relationship at baseline (*r* = 0.88, *p* < 0.001). This correlation remained significant at 5 min (*r* = 0.66, *p* < 0.001) but became weak and statistically non-significant at 10 min (*r* = 0.11, *p* = 0.489) and onwards.

**Graph 1:**
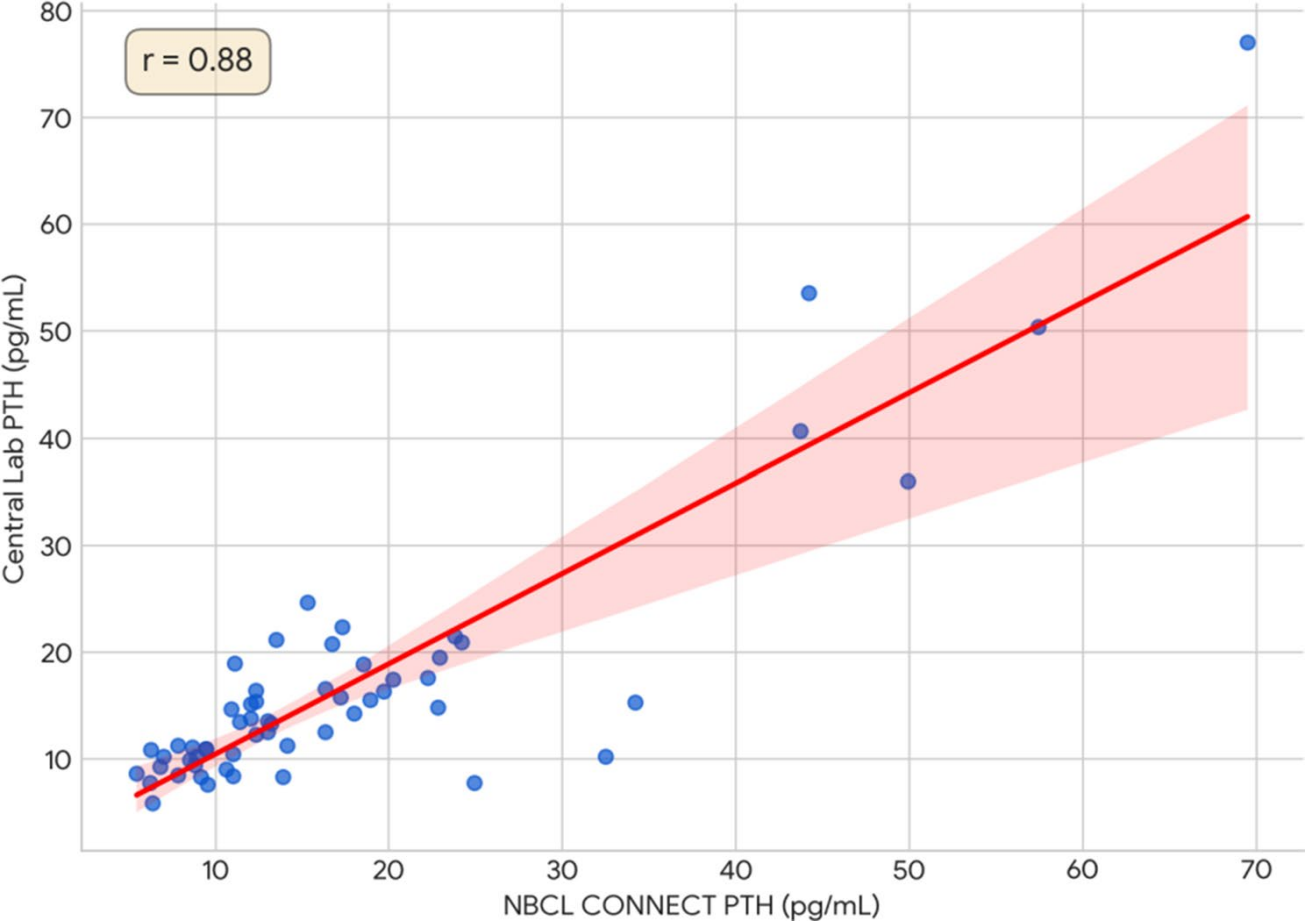
Baseline PTH correlation (Pre-Incision): scatter plot showing strong correlation (*r* = 0.88) between the NBCL CONNECT and central lab assays for baseline PTH levels, showing excellent pre-operative agreement

**Graph 2:**
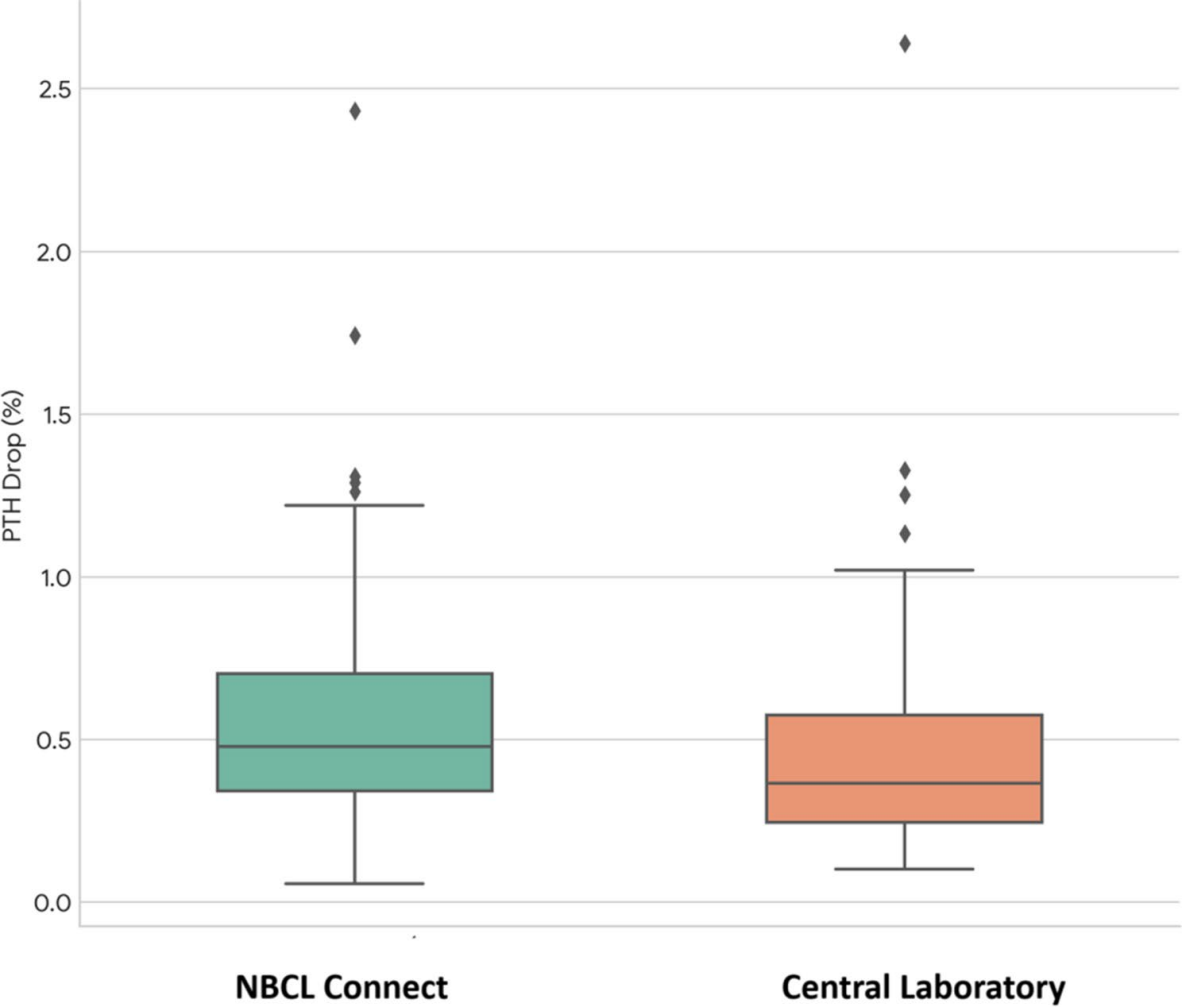
Comparison of percentage PTH drop at 5 min. The box plot shows the distribution of percentage drops measured by the NBCL CONNECT assay and the central laboratory. The NBCL assay reported a higher mean percentage drop

Bland-Altman analysis provided further insight into clinical agreement (Graph 1). Pre-incision, a minimal mean bias of 1.00 pmol/L was observed. At the 10-minute time interval, where a significant difference was noted, the mean bias was 2.21 pmol/L with markedly wide 95% limits of agreement (-14.3 to 18.8 pmol/L), which revealed a highly scattered, unpredictable relationship between the methods. Scatter plots comparing the methods against the line of identity visually confirmed these findings (Graph 2).

### Discordant results

**Graph 3:**
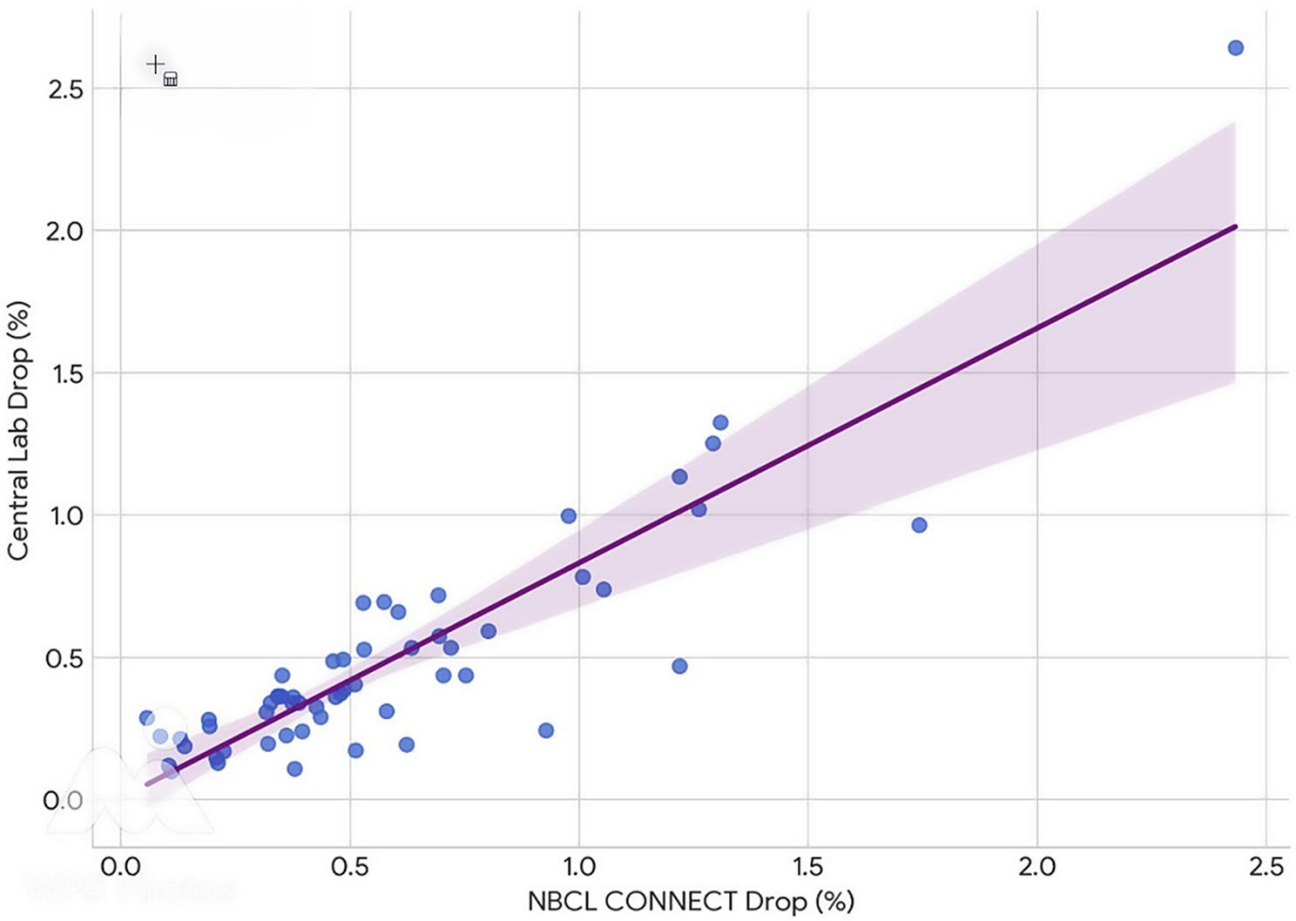
Correlation of the percentage drops at 5 min: scatter plot showing strong correlation between the percentage drops measured by both assays at 5 min, indicating high consistency in assessing the relative PTH decay

An analysis of discordant outcomes revealed a false positive rate of 7.0% (4 cases) and the same number for false negative results, when looking at the NBCL assay. Only one of these four patients was a failed surgery and required further operation (missed multiglandular disease). The other three cases had microclots in the blood bottle, which we were informed of by the POC testing coordinator after the surgery was performed, as these three samples showed a false positive on lab testing (Roche Cobas) as well (Graph 3).

For the four false negative results using NBCL (7%), a common theme among these patients was a low baseline PTH. This issue appears to be secondary to interference.

## Discussion

This study provides a robust statistical comparison of a POC PTH assay (NBCL) and a standard laboratory method during parathyroid surgery. The two methods do not show any statistically significant difference at baseline or 5 min post-excision, but there is a divergence which occurs at 10 and 15 min, making the test less reliable for intraoperative decision-making as the time post excision increases, which is consistent with other studies on the POC devices [2, 8].

The combination of a statistically significant bias (from the Wilcoxon test), a breakdown in correlation (*r* = 0.11), and wide limits of agreement revealed by the Bland-Altman analysis, indicates an unreliable and unpredictable relationship between the assays taken at and after the 10 min interval. Surgeons relying on the POC assay after 10 min post gland excision may be making decisions based on data that does not accurately reflect the results from the laboratory standard.

Our results are in line with a recent multicentre study on the NBCL CONNECT system, validating its use in parathyroid surgeries [9]. However, our study provides a novel contribution in the quantitative analysis of the percentage drop itself. While previous studies have focused on the overall accuracy and correlation, our data demonstrates that the NBCL CONNECT assay reports a statistically significant and larger percentage drop than the central laboratory standard [2, 9]. This finding has direct clinical implications for the application of the Miami criterion and represents a new & nuanced understanding of this specific POC device’s behaviour.

Crucially, our analysis highlights that surgeons must be prepared for both types of discordant results. The false-positive cases underscore classic pitfalls of IOPTH-guided surgery, such as missed multiglandular disease, incorrect gland identification or clotting in the blood bottle, where the PTH drop can be misleading [2].

Conversely, the false-negative cases demonstrate situations where a lack of a >50% drop does not necessarily mean surgical failure. Our finding that these cases often had relatively low baseline PTH levels suggests the PTH decay kinetics may be different in this subgroup. This may be due to factors such as the presence of different PTH fragments in circulation, which can interfere with some assays more than others, a known pitfall of IOPTH monitoring [10]. This emphasises that IOPTH results must be interpreted in the full clinical context.

Unlike studies that may focus primarily on one failure mode, our review of both false-positive and false-negative cases provides a more complete clinical picture of the assay’s real-world limitations. By identifying common characteristics of these discordant cases, we offer valuable insights that can help guide a surgeon’s judgement when a test result is equivocal.

This study has limitations that we acknowledge. Firstly, as this was a single-center study, our findings may reflect local practices and patient populations that may not be generalisable to all surgical units. Secondly, while the overall cohort size is robust, the subgroup analyses of discordant results are based on a small number of false-positive and false-negative cases, meaning the conclusions drawn from these specific groups should be considered exploratory and will benefit from further studies.

We got in touch with the NBCL representative on account of a few instances when the NBCL IOPTH assay did not show a drop, but the standard laboratory testing method did (false negative results), and the histology later confirmed the excised specimen to be an adenoma. We were informed that other hospitals in Europe have also noticed something similar in a few cases. The reasoning and explanation given to us, after NBCL analysed the sent samples, was that it is attributable to the rheumatoid factors present in specific patients which occurs very infrequently, affecting less than 1% patients across Europe, who had parathyroid surgeries utilising the NBCL IOPTH analyser. This phenomenon has been reported in the literature. Cavalier et al., in a series of 734 patients with elevated PTH levels, reported that 3.4% presented interference due to heterophilic antibodies and 1.2% presented interference due to rheumatoid factor [11].

We acknowledge that the routine use of near-infrared autofluorescence (NIRAF) with the Fluobeam^®^ system in all cases is an additional tool that contributes to the success of the surgery. The specific contribution of NIRAF cannot be disentangled from that of the IOPTH assay in this study design, a factor that should be considered when interpreting the overall outcomes.

## Conclusion

The NBCL CONNECT point-of-care assay is an efficient tool for IOPTH monitoring, but it is not a replacement for the standard laboratory method. Its usefulness lies in interpreting the drop in a parathyroid adenoma excision surgery, where a greater than 50% drop in PTH using the POC assay is a marker of success of the surgical procedure, the absolute values themselves are not to be used as results. While showing reasonable correlation at baseline, the methods diverge as PTH level falls. Clinicians should exercise caution when using this POC assay for critical intraoperative decisions, particularly after 10 min post-gland excision, where the agreement with the laboratory standard becomes unreliable. Furthermore, the potential for both false-positive and false-negative results necessitates that the data be used as an adjunct to, and not a replacement for sound surgical judgement.

## Data Availability

All raw data generated and analysed during the current study is already included in this published article. The data analysis files (statistical scripts and processed data) are not publicly available as they contain intermediate identifiers or institutional information but are available from the corresponding author on reasonable request.
